# *Cortisol total/CRP* ratio for the prediction of hospital-acquired pneumonia and initiation of corticosteroid therapy in traumatic brain-injured patients

**DOI:** 10.1186/s13054-019-2680-6

**Published:** 2019-12-05

**Authors:** Marwan Bouras, Antoine Roquilly, Pierre-Joachim Mahé, Raphaël Cinotti, Mickaël Vourc’h, Bastien Perrot, Kalyane Bach-Ngohou, Damien Masson, Karim Asehnoune

**Affiliations:** 1 Surgical Intensive Care Unit, Hotel-Dieu, University Hospital of Nantes, 44093 Nantes, France; 2 EA3826 Therapeutiques Anti-Infectieuses, Institut de Recherche en Sante 2 Nantes Biotech, Medical University of Nantes, 44000 Nantes, France; 3 UMR_S 1246 Methods in Patient-Centered Outcomes and Health Research, Nantes University, 44000 Nantes, France; 4 Biochemistry Laboratory, UMR INSERM 1235, University Hospital of Nantes, 44093 Nantes, France; 5 Department of Anesthesia and Critical Care, Hôtel Dieu, University Hospital of Nantes, 1 place Alexis Ricordeau, 44093 Nantes Cedex 9, France

**Keywords:** Traumatic, Brain, Injury, Pneumonia, Cortisol, CRP, Corticosteroid, Biomarkers

## Abstract

**Background:**

To propose a combination of blood biomarkers for the prediction of hospital-acquired pneumonia (HAP) and for the selection of traumatic brain-injured (TBI) patients eligible for corticosteroid therapy for the prevention of HAP.

**Methods:**

This was a sub-study of the CORTI-TC trial, a multicenter, randomized, double-blind, controlled trial evaluating the risk of HAP at day 28 in 336 TBI patients treated or not with corticosteroid therapy. Patients were between 15 and 65 years with severe traumatic brain injury (Glasgow coma scale score ≤ 8 and trauma-associated lesion on brain CT scan) and were enrolled within 24 h of trauma. The blood levels of CRP and cortisol_total&free,_ as a surrogate marker of the pro/anti-inflammatory response balance, were measured in samples collected before the treatment initiation. Endpoint was HAP on day 28.

**Results:**

Of the 179 patients with available samples, 89 (49.7%) developed an HAP. Cortisol_total&free_ and CRP blood levels upon ICU admission were not significantly different between patients with or without HAP. The cortisol_total_/CRP ratio upon admission was 2.30 [1.25–3.91] in patients without HAP and 3.36 [1.74–5.09] in patients with HAP (*p* = 0.021). In multivariate analysis, a cortisol_total_/CRP ratio > 3, selected upon the best Youden index on the ROC curve, was independently associated with HAP (OR 2.50, CI95% [1.34–4.64] *p* = 0.004). The HR for HAP with corticosteroid treatment was 0.59 (CI95% [0.34–1.00], *p* = 0.005) in patients with a cortisol_total_/CRP ratio > 3, and 0.89 (CI95% [0.49–1.64], *p* = 0.85) in patients with a ratio < 3.

**Conclusion:**

A cortisol_total_/CRP ratio > 3 upon admission may predict the development of HAP in severe TBI. Among these patients, corticosteroids reduce the occurrence HAP. We suggest that this ratio may select the patients who may benefit from corticosteroid therapy for the prevention of HAP.

## Introduction

Traumatic brain injury (TBI) is the leading cause of mortality and disability among young patients throughout the world. It is a major health and socioeconomic problem [1, 2]. Hospital-acquired pneumonia (HAP), whose incidence ranges from 40 to 60% for severe traumatic brain-injured patients [3], is associated with poor neurologic outcome and death [4]. TBI induces a disturbance of the normally balanced interplay between pro-inflammatory and anti-inflammatory mechanisms leading to a greater susceptibility to infections including HAP [5].

During the early post-traumatic period, the release of danger-associated molecular pattern by injured cells results in systemic inflammatory response syndrome (SIRS) which is characterized by increased levels of CRP and cytokines [6–8]. Elevated blood concentrations of CRP upon ICU admission are correlated with an increased risk of organ failure and death [9] and persistent systemic inflammatory response syndrome is predictive of hospital-acquired infection in trauma patients [10]. To avoid the dramatic consequences of an overwhelming SIRS such as organ failure, an anti-inflammatory response (called "compensatory anti-inflammatory response syndrome" : CARS) is rapidly triggered by the host, including cortisol secretion by adrenal glands after afferent impulses from the site of injury [11].

It is true that the administration of corticosteroids in a condition that is at risk of secondary infection may seem inappropriate. However, we have shown that low dose of hydrocortisone prevents the occurrence of hospital-acquired pneumonia in multiple trauma patients [12]. The anti-inflammatory properties of corticosteroids reduce lung inflammation secondary to trauma and enhance the functions of immune cells like dendritic cells and thus could limit secondary bacterial pneumonia [13]. The administration of corticosteroids in a context of relative immunosuppression shown after any acute condition is counter intuitive but low-dose corticosteroids may enhance immunity. Indeed, low-dose hydrocortisone improves the phagocytic abilities of neutrophils, decreases the blood concentration of anti-inflammatory cytokines such as interleukin-10, and increases the blood concentrations of interferon-γ and interleukin-12, cytokines involved in the host defense against infections [14, 15].

We aimed to assess the predictive values of the cortisol/CRP ratio (surrogate marker of the CARS/SIRS balance) for the development of HAP in TBI patients. We also evaluated if this ratio may help to select patients who would benefit from corticosteroid therapy to prevent HAP.

## Materials and methods

### Study design

This study is a sub-study of the Corti-TC trial [3] (NCT 01093261) a multicentre, randomized, double-blind, placebo-controlled trial of hydrocortisone and fludrocortisone in severe traumatic brain injury. Patients admitted in 19 French ICUs were enrolled in the Corti-TC trial from Sept. 1, 2010, to Nov 29, 2012. Prior to enrollment, written informed consent was obtained from a next-of-kin. Retrospective consent was obtained from patients when it was possible.

### Patients

In the Corti-TC trial, inclusion criteria were age between 15 and 65 years, severe traumatic brain injury (Glasgow coma scale score ≤ 8 and trauma-associated lesion on brain CT scan), and enrolment within 24 h of trauma [16, 17]. Exclusion criteria were as follows: treatment with corticosteroids in the previous 6 months, immunosuppression, pregnancy, tetraplegia, or antibiotic treatment at the time of inclusion. In this sub-study of the Corti-TC trial (*n* = 330 patients), we included the 179 patients with available blood samples.

### Corticosteroid therapy

For the purpose of the Corti-TC trial, patients received either hydrocortisone (200 mg per day tapered) and fludrocortisone (50 μg tablet once per day) or double placebo for 10 days. Before receiving study drug, adrenal function was assessed with a short corticotropin test. Treatment was stopped if patients had no adrenal insufficiency which was defined as basal blood cortisol concentration of less than 150 μg/L (413 nmol/L) or a maximum increase of less than 90 μg/L (248 nmol/L) in the 60 min after a short corticotropin test.

### Endpoints

In the Corti-TC study, the primary outcome was hospital-acquired pneumonia on day 28 of follow-up in patients with or without corticosteroid therapy. In this sub-study, the primary outcome was also the rate of hospital-acquired pneumonia in severe traumatic brain-injured patients.

### Blood samples

Blood samples were collected in the first 24 h after trauma, before any administration of the Corti-TC treatment. Sera were frozen − 80 °C upon dosage. Concentration of CRP and plasma cortisol concentrations (free and total) were investigated in sera collected in patients from the Corti-TC study with available samples.

### Measurement of CRP, cortisol, and cortisolemia

All biochemical measurements of CRP, Transcortin, and total and free cortisol were performed at the laboratory of Clinical Biochemistry, Nantes University Hospital. The laboratory is licensed according to the ISO 15189 accreditation standard for clinical laboratories.

Serum CRP and total cortisol were determined in single measurement with an electrochemiluminescence immunoassay (C-Reactive Protein Gen.3 and Elecsys Cortisol II respectively) on the Cobas e602-module of the automated cobas®8000 system (Roche Diagnostics, Mannheim, Germany). The lower limits of detection (LoD) of the CRP and cortisol assays were 0.3 mg/L and 0.54 μg/L respectively. Each CRP and cortisol runs were validated by measuring two levels of quality control material prior to starting the experiment.

Samples for serum-free cortisol determination were prepared by equilibrating 500 μL of serum at 37 °C for 15 min in Centrifree 30,000 molecular weight cut-off Centrifugal filters (Merck Millipore, Tullagreen, Ireland) before centrifugation at 1500*g* for 30 min at 37 °C. Free cortisol was determined in the ultrafiltrate as previously describe for total cortisol.

Serum Transcortin concentrations were measured in duplicate using Human Corticosteroid Binding Globulin ELISA (BioVendor, Brno, Czech Republic) according to the manufacturer’s instructions. Each series of assays (1 ELISA plate) was validated by two internal controls. The lower limit of detection (LoD) was 0.01 μg/mL.

### Care of patients with severe TBI

All care provided to patients with severe TBI followed the international guidelines in effect at the time of randomization, including respiratory management, temperature management, stress ulcer prophylaxis, nutrition, fluid therapy, glucose management, intracranial pressure monitoring, and management [18] as done in our previous studies on the subject [19, 20].

### Hospital-acquired pneumonia (HAP) definition

Pneumonia was suspected as diagnosis when at least two of the following signs: body temperature > 38 °C; leukocytosis > 12,000/mL or leukopenia < 4000/mL; purulent pulmonary secretions; were associated with the appearance of a new infiltrate or changes in an existing infiltrate on the chest X-ray. Diagnosis was confirmed by tests on a respiratory tract sample using a quantitative culture with a predefined positive threshold of 10^4^ colony-forming units per milliliter (CFU/mL) for a broncho-alveolar lavage or nonbronchoscopic sample or 10^3^CFU/mL for a protected specimen brush. Respiratory samples were always obtained before starting any new antibiotic treatment. HAP was defined as pneumonia that occurred 48 h after admission [21]. All HAP recorded in the study where early-onset pneumonia (< 7 days).

### Data collection

Overall, patient characteristics, including demographics, injury severity score and abbreviated injury score, fluid infusions, vasopressors, antibiotic prophylaxis, CRP rates, plasma cortisol concentrations (free and total), surgery, infections, organ failures, length of ventilatory support, ICU hospitalization, and 28th day mortality, were recorded.

### Statistical analysis

Continuous data were described as median [1st–3rd quartiles] and values were compared using the Wilcoxon-Mann-Whitney test. Categorical data were described as *N* (%) and values were compared using the chi-square test or the Fisher exact test.

The cut-off for distinguishing low versus high (cortisol_total_/CRP) ratio was based on logistic regression between HAP and (cortisol_total_/CRP) ratio. The value corresponding to the largest Youden index was selected as the cut-off. Variables that were associated with HAP at the 0.15 level in univariate analysis were included in a multiple logistic regression. Then, variables that were non-significant at the 0.05 level (Wald test) were removed one by one. Multiple logistic regressions were performed in the global sample, in the placebo group and in the corticosteroid group.

## Results

### Patients

Of the 179 analyzed patients, 89 (49.7%) developed a HAP and 90 (50.3%) did not. Table 1 shows the baseline characteristics. All cases of recorded HAP were ventilator-associated pneumonia. There were no differences in the demographics of patients (including severity scores) who developed HAP compared with those who did not develop HAP except for the body temperature > 39 °C (20% vs 5.6% respectively *p* = 0.004). Patients were equally drawn from the intervention and control arms and they do not differ in other important ways from the original study population in terms of age, sex, severity, and comorbidities (data not shown).

**Table 1:** Baseline characteristics

	Hospital-acquired pneumonia, no	Hospital-acquired pneumonia, yes	*P* value
	*N* = 90	*N* = 89	
Age, years	34 [23–48]	31 [23–48]	0.83
Men	72 (80)	74 (83.2)	0.58
Medical history, No. (%)			
Renal insufficiency	0 (0.00)	1 (1.12)	0.49
Cardiac insufficiency	1 (1.11)	1 (1.12)	1.00
Chronic pulmonary disease	1 (1.11)	2 (2.25)	0.62
Pathological admission status, median			
SAPS II	42 [34–50]	42 [37–50]	0.29
Injury Severity Score	18 [9–29]	24 [13–30]	0.10
Glasgow coma scale	6 [4–7]	6 [4–7]	0.85
Associated Thoracic trauma (AIS ≥ 3)	24 (26.67)	29 (32.58)	0.38
Events prior to admission, No. (%)			
Hemorrhagic shock	5 (5.56)	5 (5.62)	0.98
Hypotension (systolic arterial pressure < 120 mmHg)	24 (26.67)	24 (26.97)	0.96
Neurosurgery	29 (32.22)	22 (24.72)	0.26
Etomidate use	62 (68.89)	64 (71.91)	0.65
Blood transfusion			
Red cell units	29 (32.22)	21 (23.60)	0.19
Frozen plasma units	29 (32.22)	23 (25.84)	0.34
Systemic inflammatory response syndrome upon admission			
Cardiac rate > 120/min	16 (17.78)	13 (14.61)	0.56
PaCO2, mmHg	38 [33–41]	37 [33–43]	0.86
Body temperature > 39.0 °C	18 (20)	5 (5.6)	0.004
Leucocytes count (g/L)	11.9 [7.8–15.9]	13.4 [10.3–16.5]	0.06
Neutrophil count (g/L)	8.4 [6–15.5]	10.6 [8–14.5]	0.19
Lymphocyt count (g/L)	1.3 [0.8–2.2]	1.2 [0.8–1.8]	0.58
C-reactive protein (mg/mL)	61 [35–98]	53 [33–82]	0.20
Short corticotropin test results			
Baseline blood cortisol_total_ concentration, μg/L	143 [92–201]	168 [111–225]	0.06
Baseline blood cortisol_free_ concentration, μg/L	16.6 [6.6–39]	21.9 [9.6–41]	0.26
Transcortin blood level, μg/mL	21.9 [14.9–47.3]	27 [16.5–51.4]	0.12
Delta total cortisolemia after 60 min after corticotropin, μg*/*L	98 [30–161]	84 [33–147]	0.89
Ratio inflammatory response/cortisol response			
Cortisol_free_/CRP	0.25 [0.08–0.71]	0.394 [0.17–0.80]	0.10
Cortisol_total_/CRP	2.30 [1.25–3.91]	3.36 [1.74–5.09]	0.021
Total Delta 60 min cortisol/CRP	1.45 [0.51–2.73]	1.55 [0.59–3.30]	0.65

*CRP* C reactive protein. Results expressed as median (1st-3rd quartile) or *N* (%), *SAPS II* Simplified Acute Physiological Score, *AIS* Abbreviated Injury Scale

### Cortisol function and CRP levels

Despite a trend for higher total and free blood levels of cortisol upon ICU admission, there was no significant difference between patients with or without HAP (*p* = 0.06 and *p* = 0.26 respectively, Table 1). The delta of cortisolemia at 60 min was not different between the two groups (98 [30–161] and 84 [33–147]; *p* = 0.89). The blood level of CRP was 61 [35–98] mg/L in patients without HAP as compared to 53 [33–82] mg/L in patients with HAP (*p* = 0.20). These results demonstrate that taken separately, blood levels of cortisol and of CRP cannot predict HAP. We thus aimed to investigate the prognostic value of a ratio combining markers of CIRCI and inflammatory response.

### Combination of cortisol_free_, cortisol_total_, and CRP to predict the development of HAP

**Fig. 1:**
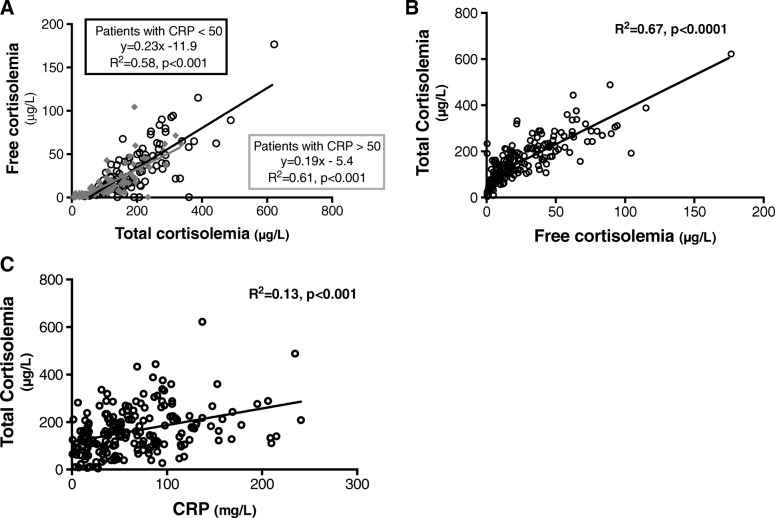
Correlations between cortisol_free_ and cortisol_total_ in high (CRP > 50 mg/mL) and low (CRP < 50 mg/mL) inflammatory patients (**a**), between cortisol_free_ and cortisol_total_ (**b**), and between cortisol_total_ and CRP blood level (**c**)

The correlation between cortisol_free_ and cortisol_total_ was consistent in spite of low or high CRP blood levels (Fig. 1a) when cortisol_free_ and cortisol_total_ were strongly correlated (Fig. 1b) suggesting that these two values can be used in patients with or without severe inflammatory response. Cortisol_total_ was correlated with blood level of transcortine (Additional file 1: Figure S1). Transcortine is the major transport protein for glucocorticoids and approximately 85% of the cortisol in circulation is bound to transcortin. We also decided to use cortisol_total_ for subsequent analysis of the risk of HAP also because it is more available easily in clinical practice. The blood level of cortisol_total_ increased with CRP (Fig. 1c), and the blood level of CRP alone was not able to discriminate patients at risk to develop HAP (Additional file 1: Figure S2). However, the cortisol_total_/CRP ratio was 2.30 [1.25–3.91] in patients without HAP and 3.36 [1.74–5,09] in patients with HAP (*p* = 0.021) (Table 1 and see Additional file 1: Figure S3). These results suggest that the combination of cortisol_total_ and CRP could help to predict HAP.

### The cortisol_total_/CRP ratio is an independent risk factor for HAP

**Fig. 2:**
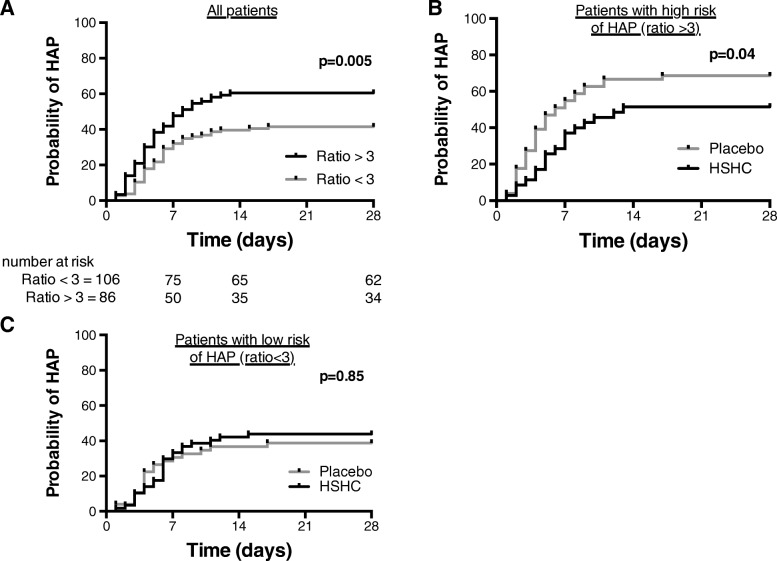
Kaplan-Meier curves for hospital-acquired pneumonia for the entire population (**a**), in patients with high risk of HAP (ratio cortisol_total_/CRP > 3) (**b**), and in patients with low risk of HAP (ratio cortisol_total_/CRP < 3) (**c**)

In an attempt to simplify the use of the results in clinical practice, the cortisol_total_/CRP ratio was dichotomized in two categories (above or below a threshold). The cut-offs of cortisol_total_/CRP ratio > 3 was selected because it provides the best sensitivity/specificity balance (ROC curves; Additional file 1: Figure S4) and the best calibration of the multivariate analysis (Hosmer–Lemeshow test *p* value > 0.05). The positive likelihood ratio was 1.66 for a cortisol_total_/CRP ratio > 3. In the entire population, patients with a ratio total cortisol/CRP *>* 3 (*n* = 88) developed more HAP than those with a ratio < 3 [50 (62.50%) vs 39 (39.39%) respectively *p* = 0.002] (Table 3). The Kaplan-Meier estimator for HAP at day 28 was 41.5% in the low ratio group (< 3) and 60.5% in the high ratio group (> 3), HR = 0.55 [(95% CI 0.36 to 0.84)] *p* = 0.005 (Fig. 2a). In multivariate analysis, a ratio cortisol_total_/CRP > 3 was independently associated with HAP (OR 2.50, 95% CI (1.34–4.64) *p* = 0.004) (Table 2). In the entire population, there was no difference in terms of mean duration of mechanical ventilation, ICU length of stay, or in-ICU mortality between the corticosteroid-treated patient group and the placebo group (Table 3). However, this ratio also discriminates the number of antibiotic-free days with a higher “antibiotics free days at day 28” in the “low ratio” group: (21d [16–26] vs 18.5d [11–21] *p* = 0.007).

**Table 2:** Multivariate analysis of the factors associated with of hospital-acquired pneumonia (*N* = 179)

	Odds ratio (95% confidence interval)	*P* value
Body temperature > 39.0 °C	0.25 [0.09–0.72]	0.010
Cortisol_total_/CRP > 3	2.50 [1.34–4.64]	0.004

**Table 3:** Comparison of outcomes of patients with high or low *(*cortisol_total_/CRP) ratio

	Entire population			Placebo				Hydrocortisone treatment	
	Low (cortisol_total_/ CRP) ratio	High (cortisol_total_/CRP) ratio	*P* values	Low (cortisol_total_/CRP) ratio	High (cortisol_total_/CRP) ratio	*P* values	Low (cortisol_total_/CRP) ratio	High (cortisol_total_/CRP) ratio	*P* values
Hospital-acquired pneumonia	39 (39.39)	50 (62.50)	0.002	18 (37.50)	32 (65.31)	0.006	21 (41.18)	18 (58.06)	0.138
Pa02/Fi02 ratio	146.5 [98–217]	175.5 [133–232]	0.147	164.5 [130–217]	175.5 [133.5–246]	0.581	122 [78–232.5]	174 [132–227]	0.156
Septic shock	5 (12.82)	2 (4.00)	0.233	2 (11.11)	0	0.125	3 (14.29)	2 (11.11)	1.00
Other infections									
Meningitis	2 (2.02)	1 (1.25)	1.000	1 (2.08)	0	0.495	1 (1.96)	1 (3.23)	1.000
Urinary tract infection	9 (9.09)	11 (13.75)	0.325	3 (6.25)	10 (20.41)	0.041	6 (11.76)	1 (3.23)	0.245
Bacteremia	8 (8.08)	5 (6.25)	0.639	4 (8.33)	3 (6.12)	0.715	4 (7.84)	2 (6.45)	1.000
Organ failures									
Duration of vasopressor support, days	4 [3–6]	4 [2–6]	0.925	4 [3–6]	5 (2–6]	0.837	4 [3–5.5]	4 [2–6]	0.787
Acute respiratory distress syndrome	18 (18.18)	16 (20.00)	0.758	5 (10.42)	9 (18.37)	0.265	13 (25.49)	7 (22.58)	0.766
Acute kidney injury	0	1 (1.25)	0.447	0	0	–	0	1 (3.23)	0.378
SOFA day 1	9 [8–10]	9 [7–10]	0.592	9 [8–9.5]	9 [7–10]	0.877	9 [7–10]	9 [7–10]	0.511
SOFA day 3	8 [6–9]	8 [5.5–9]	0.706	8 [6–9]	7 [5–9]	0.383	8 [6–9]	8 [6–10]	0.658
SOFA day 7	4 [2–7]	5 [4–6]	0.398	4 [2.5–6.5]	4 [4–6]	0.352	5 [1–8]	5 [4–6]	0.810
Outcomes									
Antibiotic-free days at day 28	21 [16–26]	18.5 [11–21]	0.007	21.5 [15.5–26.5]	19 [12–21]	0.225	21 [17–25]	18 [11–22]	0.52
Mean duration of mechanic ventilation (days)	11 [7–20]	13 [8–19.5]	0.725	11.5 [7.5–18]	12 [8–22]	0.525	11 [7–25]	13 [9–19]	0.86
Mean duration of intensive care (days)	15 [10–24]	17 [11–28]	0.288	16 [11–22]	17 [12–28]	0.167	15 [9–29]	17 [10–28]	0.867
Death in ICU									
In-ICU death	8 (8.01)	9 (11.25)	0.472	2 (4.17)	5 (10.20)	0.436	6 (11.76)	4 (12.90)	1.000

### The cortisol_total_/CRP ratio may predict the patients who will benefit from corticosteroid therapy

Finally, we tested the hypothesis that the ratio cortisol_total_/CRP has the potential to select patients who will benefit from corticosteroid therapy, and those whose risk of HAP will not be decreased by the treatment. We thus compared the relative risk of HAP in the low and the high ratio groups, according to the treatment received (placebo or corticosteroid therapy). In high-risk patients (ratio > 3), the probability of HAP at day 28 was higher in the placebo group vs corticoid group (68.6% vs 51.4% respectively, *p* = 0.04) with an hazard ratio of 0.59 (CI95% [0.34–1.00], *p* = 0.005) (Fig. 2b). Patients with a ratio > 3 were comparable on key demographic characteristics in the corticosteroid and placebo groups (Additional file 1: Figure S5). In patients with low cortisol_total_/CRP (< 3), there was no difference between placebo and treatment groups for the development of HAP (HR = 0.89 (CI95% [0.49–1.64], *p* = 0.85)) (Fig. 2c). We conclude that the protective effect of corticosteroid in TBI patients for the risk of HAP was found mainly in the subgroup of patients with a cortisol_total_/CRP ratio > 3.

## Discussion

In this sub-study of Corti-TC trial [3], we aimed to establish a correlation between the ratio “inflammatory response”/CARS and the occurrence of HAP in head trauma patients. After a TBI, pro-inflammatory cytokine secretion is a physiologic process which aims to induce damaged tissues healing and anti-bacterial activity by activating both innate and adaptive immunity. In order to balance an excessive pro-inflammatory response, the CNS induces an important anti-inflammatory response leading to increased susceptibility to infections [5]. This anti-inflammatory response is mediated by the sympathetic nervous system [22], parasympathetic nervous system [23], and the hypothalamic-pituitary system via glucocorticoid secretion. In critically ill patients, reduced cortisol breakdown contributes to abnormal blood cortisol levels [24]. This phenomenon, called Critical Illness-Related Corticosteroid Insufficiency (CIRCI), corresponds to the impairment of the hypothalamic-pituitary axis (HPA) during critical illness resulting from inadequate anti-inflammatory response for the severity of a given patient [25].

In order to correct the post-traumatic immunosuppression, many therapies have been tested in recent years. They aimed either to limit the initial SIRS (and thus the CARS) in particular by the use of low-dose glucocorticoids [3, 12] or to restore the secretion of pro-inflammatory cytokines by the use of IFN-γ, GM-CSF [26], or interleukin 12 [27]. In a multicenter, randomized, double-blind, placebo-controlled trial, Torres et al. showed that among patients with severe community-acquired pneumonia and high initial inflammatory response (CRP > 150 mg/L), methylprednisolone (0.5 mg/kg/12 h) compared with placebo decreased treatment failure [28]. In major trauma patients, CIRCI occurs frequently and is associated with uncontrolled inflammatory response, longer vasopressors infusion and poor outcomes [29]. In a large randomized trial in multiple trauma patients, we found that hydrocortisone therapy prevented the development of hospital-acquired pneumonia by day 28 in patients with CIRCI (defined by a change in baseline cortisol at 60 min of < 9 μg/dl after ACTH (250 μg) administration) [12]. However, in head trauma patients, we found no interaction between response to corticosteroid therapy and CIRCI status (using the same definition as previously described) [3]. The actualized recommendations for the diagnosis of CIRCI provide that ACTH stimulation test was not superior to random cortisol for the routine diagnosis of CIRCI [30]. Moreover, measuring plasma-free cortisol level over plasma total cortisol level was not recommended in patients with suspected CIRCI [30]. Here, the total and free cortisol blood levels were strongly correlated independently of the inflammatory status of the patient, explaining why we choose to focus on total cortisolemia.

In our study, the correlation between total cortisol and CRP levels could be explained by the early secretion of interleukin-6 (IL-6) following trauma. Indeed, IL-6 is a pro-inflammatory cytokine known to have HPA-activating activity independent of ACTH. Thus, human IL-6 increases plasma concentrations cortisol in mice [31] and during immune system activation, such as post-traumatic inflammation or septic shock. In head-injured children, serum IL-6 and CRP levels are elevated and correlated to the severity of head trauma [32] and increased levels of IL-6 in the early phase of severe acute traumatic brain injury is associated with the high inflammatory response such as development of ARDS [33].

In association with appropriate antibiotherapy, methylprednisolone administration was also associated with a faster reduction in blood IL-6 and CRP levels in the first 24 h of treatment of community-acquired pneumonia [34]. Before initiation of glucocorticoid therapy, basal cortisol level is positively correlated with IL-6. Corticosteroids reduce the production of IL-6 and the migration of inflammatory cells into the alveolar space leading to avoid an overwhelming inflammatory response. In patients with systemic autoimmune disease, which leads to an inflammatory state, the introduction of glucocorticoid reduced the IL-6 level and contribute to the apparent suppression of the HPA axis [35]. Among patients with traumatic brain injury, IL-6 is correlated with inflammatory states, high CRP rates, and the occurrence of HAP [36].

A recent meta-analysis of corticosteroids in pneumonia found that hydrocortisone was not useful in this context, and only prednisone or methyl prednisolone was beneficial [37]. However, this meta-analysis considered community-acquired pneumonia (CAP) rather than HAP or ventilator-acquired pneumonia (VAP). The micro-organisms involved in each entity are different; in CAP, they are frequently virulent and transmitted by inhaled aerosols. Moreover, respiratory physiology and immunity is severely impaired in patients suffering from HAP or VAP and admitted in ICU. Indeed, mechanical ventilation promotes a specific histological pattern of pneumonia [38, 39]. Furthermore, comparative analysis of the host response to CAP and to HAP in patients with critical illness has revealed distinct transcriptional and plasma protein responses [40] showing the functional alterations of the immune response in patients admitted to hospital.

Here, taken separately, pro- and anti-inflammatory biomarkers (CRP and cortisol respectively) failed to predict the development of HAP. However, patients with high cortisol_total_/CRP ratio (> 3) have a higher susceptibility to develop HAP and for these patients, the introduction of low dose of corticosteroids is able to reduce this susceptibility. This effect of corticosteroid therapy is not found in patients with a ratio < 3**.** There are two means by which low-dose corticosteroids could decrease the rate of secondary pneumonia. Firstly, through an anti-inflammatory effect (patients with a low cortisol/CRP ratio) that decrease the excessive inflammatory response and therefore the compensatory CARS response (immunosuppression). The initiation of corticosteroid therapy may for example reduce IL-6-dependent HPA stimulation, limiting the anti-inflammatory response and thus the prevalence of HAP in highly inflammatory patients. Secondly, corticosteroids may also directly enhance immunity (patients with a high cortisol/CRP ratio). Indeed, low-dose hydrocortisone improves the phagocytic abilities of neutrophils, decreases the blood concentration of anti-inflammatory cytokines such as interleukin-10, and increases the blood concentrations of interferon γ and interleukin-12, cytokines enhancing immunity and involved in the host defense against infections [14]. Glucocorticoids modulate dendritic cells during and after inflammation [13] allowing less tissue damage and therefore less sensitivity to bacterial infections. In septic shock or in viral pneumonia, glucocorticoids restore major histocompatibility complex class II expression on myeloid cells, suggesting a better antigen presentation by antigen-presenting cells during treatment [41, 42]. We and other groups have shown that hydrocortisone enhances immunity in the context of any acute immunosuppressive condition like severe trauma or sepsis [14, 15]. More specifically, we have demonstrated that trauma-induced immunosuppression is characterized by an interleukin-10-dependent elimination of dendritic cell by natural killer cells and that hydrocortisone improves outcome by limiting this immunosuppressive feedback loop [15].

The main strength of this ancillary study is the data from a randomized, multicentre, double-blind, controlled trial. This is the first study discriminating head injured patients at risk for developing pneumonia by using an easy-to-use anti- and pro-inflammatory factor ratio in common practice. Another strength of our study is the safety of low-dose corticosteroid use in trauma patients. Indeed, by closely monitoring patients’ natremia and glycemia, there are no serious adverse events recorded in the two large randomized trials we have conducted on the field [3, 12]. The use of low-dose corticosteroids therefore seems to us to be safe in patients suffering from severe TBI. However, high-dose corticosteroids are not recommended in this context because they provide serious safety issues [43]. Some limitations must be noted; first, it is impossible to know whether the effect of hydrocortisone is due to the restoration of post-inflammation homeostasis or the correction of the initial CIRCI although the use of a combination of a pro- and anti-inflammatory factor suggests that this effect is due to the correction of the post-inflammation disorder. However, the dosage of other more specific factors, such as IL-6 or IL-10, may help to refine the diagnosis of patients at risk of post-traumatic stress disorder. Second, this ratio could be refined by adding other factors such as the trauma severity score (Glasgow score) or other objective variables (gender, age, medical history), but more patients are needed to implement such a score. Third, to be validated, this ratio must be the subject of a randomized controlled trial comparing management of HAP prevention by corticosteroid therapy in patients at risk (ratio > 3). Finally, our study also suffers from insufficient evidence on secondary outcomes (duration of mechanical ventilation, ICU length of stay…). This is probably due to the size of the samples understudy and this issue could be improved by conducting a specific prospective study to validate the ratio.

## Conclusion

A cortisol_total_/CRP > 3 ratio upon admission may predict the development of HAP in patients with severe traumatic brain injury. In high-risk patients (ratio > 3), the administration of corticosteroid therapy reduces the occurrence of HAP. This ratio could be used to select patients eligible for corticosteroid therapy in prevention of HAP in patients with severe traumatic brain injury.

### Take home message

In severe traumatic brain-injured patients, the imbalance between the pro- and anti-inflammatory mechanisms leads to increased susceptibility to hospital-acquired pneumonia. We show here that cortisol and CRP blood levels together as surrogate markers of the CARS/SIRS balance upon ICU admission can accurately predict the development of hospital-acquired pneumonia and define the subgroup of patients who may benefit most from “low-dose” corticosteroid therapy.

## Supplementary information

**Additional file 1: Figure S1.** Correlations between transcortine blood level and cortisol_total_ blood level in the entire population. **Figure S2.** Receiver Operating Characteristic (ROC) curve of the risk of Hospital Acquired Pneumonia in the entire population for the CRP blood levels. **Figure S3.** Comparison of the *cortisol*_*total*_*/CRP* ratio in the entire population. Results are given in median +/- SD. **Figure S4.** Receiver Operating Characteristic (ROC) curve of the risk of Hospital Acquired Pneumonia in the entire population for the cortisol/CRP ratios. **Figure S5.** Main characteristics of patients treated with corticosteroids compared to those treated with placebo amongst the subset with ratio >3.

## Data Availability

All data and material are available in the manuscript or are available from the corresponding author on request with justification.

## Abbreviations

ACTH: Adrenocorticotropic hormone
CAP: Community-acquired pneumonia
CARS: Compensatory anti-inflammatory response
CFU: Colony-forming units
CI: Confidence interval
CIRCI: Critical illness-related corticosteroid insufficiency
CNS: Central nervous system
CRP: C-reactive protein
GM-CSF: Granulocyte-macrophage colony-stimulating factor
HAP: Hospital-acquired pneumonia
HPA: Hypothalamic-pituitary axis
HR: Hazard ratio
ICU: Intensive care unit
IL-6: Interleukin 6
IL-10: Interleukin 10
IFN- γ: Interferon γ
LoD: Limit of detection
ROC: Receiver operating characteristic
SIRS: Systemic inflammatory response syndrome
TBI: Traumatic brain injury
VAP: Ventilatory-acquired pneumonia
