# Supplementary material for: Cortisol total/CRP ratio for the prediction of hospital-acquired pneumonia and initiation of corticosteroid therapy in traumatic brain-injured patients
Source: Crit Care. 2019 Dec 5;23:394. doi: 10.1186/s13054-019-2680-6 (PMC6896691; doi:10.1186/s13054-019-2680-6)
Supplement: Supplementary file 1 — Additional file 1: Figure S1. Correlations between transcortine blood level and cortisoltotal blood level in the entire population. Figure S2. Receiver Operating Characteristic (ROC) curve of the risk of Hospital Acquired Pneumonia in the entire population for the CRP blood levels. Figure S3. Comparison of the cortisoltotal/CRP ratio in the entire population. Results are given in median +/- SD. Figure S4. Receiver Operating Characteristic (ROC) curve of the risk of Hospital Acquired Pneumonia in the entire population for the cortisol/CRP ratios. Figure S5. Main characteristics of patients treated with corticosteroids compared to those treated with placebo amongst the subset with ratio >3. [file 13054_2019_2680_MOESM1_ESM.docx]

**SUPPLEMENTAL**

S1: Correlations between transcortine blood level and cortisol_total_ blood level in the entire population.

S2: Receiver Operating Characteristic (ROC) curve of the risk of Hospital Acquired Pneumonia in the entire population for the CRP blood levels


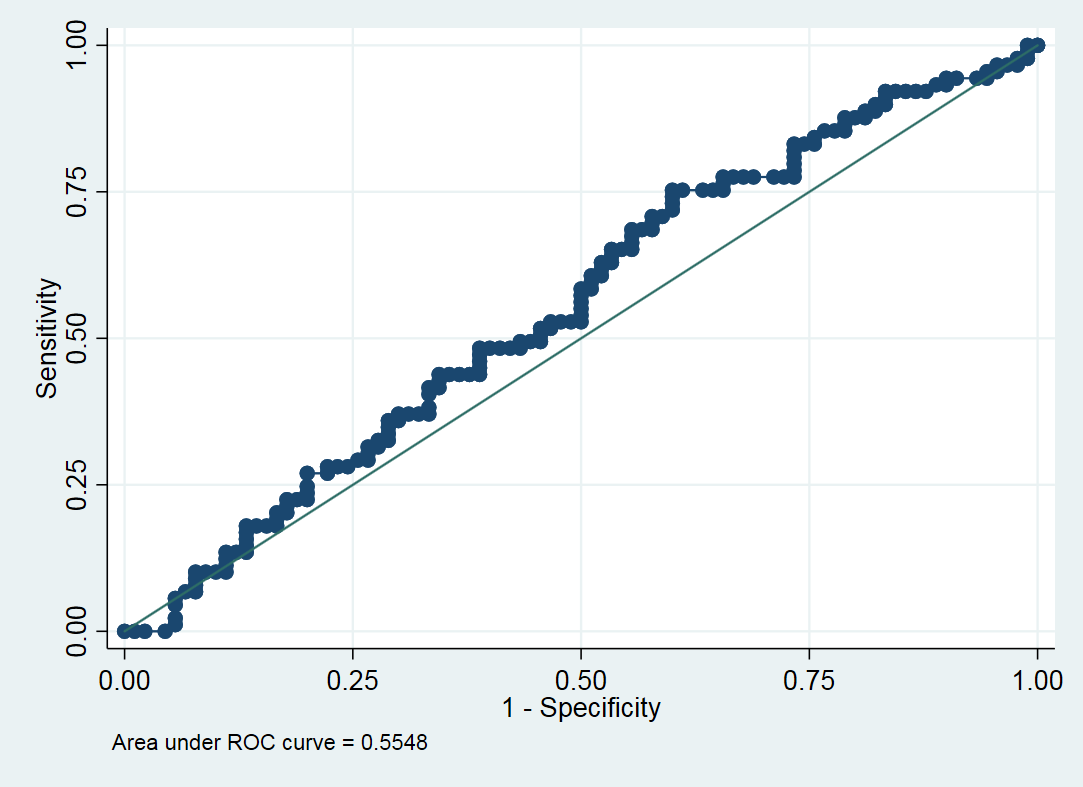


S3: Comparison of the *cortisol_total_/CRP* ratio in the entire population. Results are given in median +/- SD

S4: Receiver Operating Characteristic (ROC) curve of the risk of Hospital Acquired Pneumonia in the entire population for the cortisol/CRP ratios.


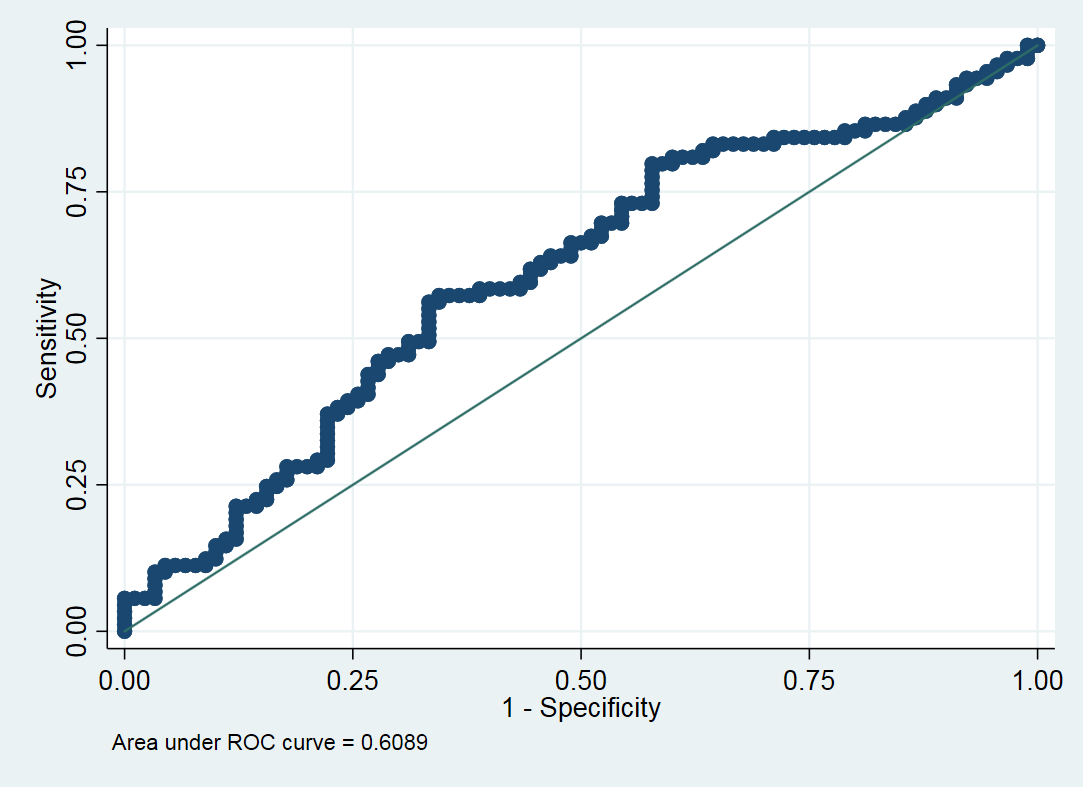


S5 : Main characteristics of patients treated with corticosteroids compared to those treated with placebo amongst the subset with ratio >3

| **Patients with ratio >3** | **Corticosteroids group** | **Placebo group** | **P-value** |  |
| --- | --- | --- | --- | --- |
|  | N=37 | N=53 |  | |
| Age, years | 32 [24- 48] | 32 [27-53] | 0.55 | |
| Men % | 78.3 | 84.9 | 0.62 | |
| ***Medical history, No. (%)*** |  |  |  | |
| Diabete | 0 (0) | 1 (1.8) | >0.99 | |
| Obesity | 1 (2.7) | 1 (1.8) | >0.99 | |
| Cardiac insufficiency | 1 (2.7) | 1 (1.8) | >0.99 | |
| Chronic pulmonary disease | 0 (0) | 0 (0) | >0.99 | |
| ***Pathological admission status, median.*** |  |  |  | |
| IGS II | 44 [38-54] | 37 [43-50] | 0.59 | |
| SOFA day 0 | 9 [7-10] | 9 [7-10] | >0.99 | |
| Glasgow Coma Scale | 6 [3-7] | 6 [3-7] | >0.99 | |
| MGAP Score | 18 [17-20] | 18 [17-20] | >0.99 | |
| Etomidate use (%) | 70,2 | 64 | 0.48 | |

Results expressed as median (1^st^-3^rd^ quartile) or N (%). SOFA =Sequential Organ Failure Assessment, MGAP= Mechanism, Glasgow coma scale, Age, and arterialPressure

IGS = simplify gravity index
